# Cultured Rat Hippocampal Neurons Exposed to the Mitochondrial Uncoupler Carbonyl Cyanide Chlorophenylhydrazone Undergo a Rapid, Presenilin-Dependent Change in Neuronal Properties

**DOI:** 10.3390/ijms25010578

**Published:** 2024-01-01

**Authors:** Liliia Kushnireva, Menahem Segal, Eduard Korkotian

**Affiliations:** 1 Faculty of Biology, Perm State University, 614068 Perm, Russia; lilikushnireva@gmail.com; 2 Department of Immunology and Regenerative Biology, The Weizmann Institute of Science, Rehovot 7610001, Israel; 3 Department of Brain Sciences, The Weizmann Institute of Science, Rehovot 7610001, Israel; eduardkorkotian@gmail.com

**Keywords:** CCCP, carbonyl cyanide m chlorophenyl hydrazone, calcium, presenilin 1, mutant presenilin 1, processes, mitochondria, Alzheimer’s disease

## Abstract

Presenilin 1 (PS1) is a transmembrane proteolytic subunit of *γ*-secretase that cleaves amyloid precursor proteins. Mutations in PS1 (mPS1) are associated with early-onset familial Alzheimer’s disease (AD). The link between mutated PS1, mitochondrial calcium regulation, and AD has been studied extensively in different test systems. Despite the wide-ranging role of mPS1 in AD, there is a paucity of information on the link between PS1 and neuronal cell death, a hallmark of AD. In the present study, we employed the selective mitochondrial uncoupler carbonyl cyanide chlorophenylhydrazone (CCCP) and compared the reactivity of mPS1-transfected cultured rat hippocampal neurons with PS1 and control neurons in a situation of impaired mitochondrial functions. CCCP causes a slow rise in cytosolic and mitochondrial calcium in all three groups of neurons, with the mPS1 neurons demonstrating a faster rise. Consequently, mPS1 neurons were depolarized by CCCP and measured with TMRM, a mitochondrial voltage indicator, more than the other two groups. Morphologically, CCCP produced more filopodia in mPS1 neurons than in the other two groups, which were similarly affected by the drug. Finally, mPS1 transfected neurons tended to die from prolonged exposure to CCCP sooner than the other groups, indicating an increase in vulnerability associated with a lower ability to regulate excess cytosolic calcium.

## 1. Introduction

Presenilins (PSs) are transmembrane proteins that regulate the cleavage of key proteins found in the plasma membrane. Among these proteins is the amyloid precursor protein (APP), that is associated with emergence of human early-onset familial Alzheimer’s disease (fAD). The clinical relevance of PS activity is illustrated by the fact that 20–50% of fAD patients carry PS mutations. This has raised great interest, as mutations in the genes that encode PSs cause the formation of amyloid plaques, a hallmark of AD. Consequently, a large body of studies has attempted to decipher the role of PS1 in the presymptomatic phases of fAD [1,2,3,4]. One of the main leads to the involvement of PS1 in fAD is their association with intracellular calcium stores of the endoplasmic reticulum (ER) and the mitochondria. It has been suggested that PSs physically link the ER with the mitochondria at specific sites called the mitochondria-associated ER membrane (MAM), where amyloid beta proteins are produced [5,6,7]. It is suggested that mPS1 affects mitochondrial functions, including the handling of excess calcium ions in the cytosol [8,9,10,11,12]. This provides a direct link to the proposed role of calcium handling machinery in triggering fAD, regardless of the direct molecular pathway associated with this link (e.g., the catalytic core of the gamma secretase [8,9]). This link was already proposed over a decade ago [9,13]. However, there is still a paucity of information on the involvement of PS1 in neuronal physiology and plasticity. A powerful tool for the analysis of the role of PS in neuronal functions is a mutant PS1 (M146V, mPS1, [9,12]). In response to caffeine or ionomycin, activators of calcium release from the ER, mPS1 causes a larger-than-normal increase in free cytosolic calcium ([Ca^2+^]_c_) [11,13,14,15]. Furthermore, spontaneous fluctuations of [Ca^2+^]_c_, which are sensitive to blockers of ER calcium release, are larger in mPS1 neurons than in controls [16]. However, Wu et al. [1] found that the conditional knockout of PS1 in mice causes a reduction in the response of cultured hippocampal neurons to caffeine, leaving this issue still unsettled. In earlier studies and in the present one, others and we [17] found that mPS1 express higher concentrations of ryanodine receptors, indicating an aberrant regulation of mitochondrial calcium, leading to cell death. Nevertheless, in the present study, we demonstrate reduced calcium signaling between the ER and mitochondria. It may indicate the failure of the attempt to compensate the signaling deficit with RYR elevation. This may produce the molecular lead to the effect of mPS1 on calcium regulation.

In a different series of investigations, cultured hippocampal neurons from PS1-M146V knock-in mice express fewer mature dendritic spines compared to control neurons [18], indicating that some synaptic functions are impaired in these neurons. Nevertheless, it is not clear how the observed morphological differences relate to mPS1 action. In more recent studies, we [19] and others [20,21] explored the effects of the selective mitochondrial uncoupler carbonyl cyanide chlorophenylhydrazone (CCCP) [22] on functional and morphological attributes of cultured neurons to describe marked and rather fast effects of the drug on mitochondrial morphology and the calcium handling ability of hippocampal dendrites in a culture. In our work [23], we suggested that the growth of filopodia from so-called “hot spots” and their further transformation into mature spines depends on the store-operated calcium entry mechanism. In a recent study, we tested the validity of this assumption [19], but the use of PS1 and mPS1 may shed a new light on the initiation of filopodia growth. We now expand these studies to compare the effects of CCCP on PS1 and mPS1 transfected neurons. We wish to report that, compared to control and PS1 transfected neurons, (1) mPS1- neurons are more sensitive to CCCP, in that their [Ca^2+^]_c_ elevates faster; (2) they cease spontaneous calcium transient discharges faster; (3) their mitochondrial potential dissipation occurs more rapidly; and (4) they are associated with a higher rate of the formation of dendritic protrusions (i.e., filopodia). This indicates that mPS1 neurons are hypersensitive to CCCP compared to the controls, leading to their earlier death.

## 2. Results

**Figure 1:**
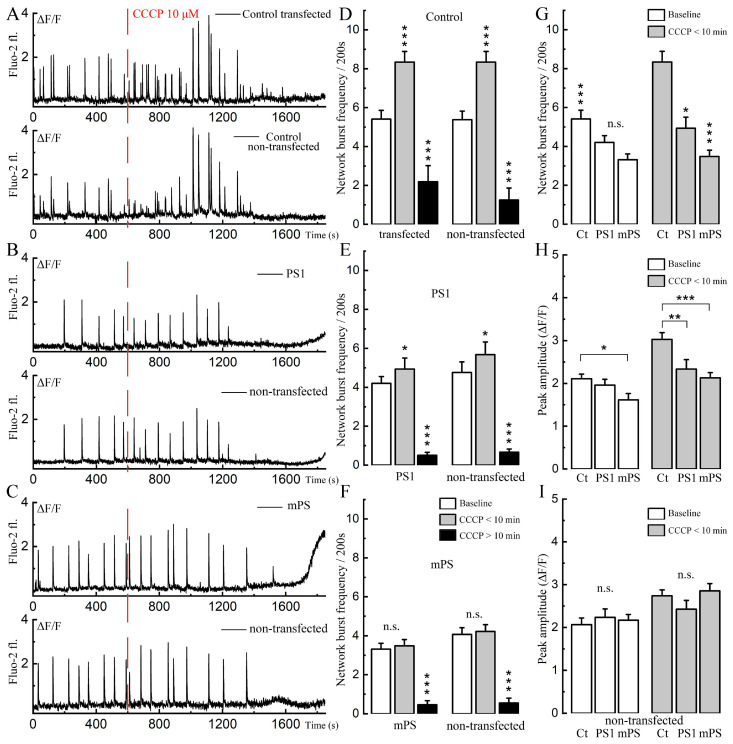
Comparison of the effect of CCCP on spontaneous calcium activity of cells transfected with normal and mutant presenilin. (**A**–**C**) Examples of spontaneous calcium activity in control, PS1-, mPS1-, and non-transfected cells for each group before and after application of 10 μM CCCP (red dotted line). (**D**–**F**) Averaged frequencies of spontaneous calcium transients (Fluo-2) per 200 s in transfected and non-transfected cells before incubation (baseline), during the first 10 min of incubation with 10 μM CCCP, and during the following 10 min; *n* = 15 cells in each group, for transfected cells: F (control) = 25.22, F (PS1) = 18.83, F (mPS1) = 16.17, three cultures, DIV 10-14. ANOVA, Bonferroni post hoc tests. (**G**) Averaged frequency of spontaneous calcium bursts per 200 s in transfected control (Ct), PS1 and mPS1 before (F = 6.8) and during the first 10 min with 10 μM CCCP (F = 24.45). (**H**) Normalized peak amplitude of spontaneous calcium bursts in transfected control (Ct), PS1 and mPS1 cells before (F = 3.96) and during the first 10 min of incubation with CCCP (F = 10.76). (**I**) Normalized peak amplitudes of spontaneous calcium events in non-transfected cells for each group before (F = 0.21) and during the first 10 min of incubation with CCCP (F = 1.98). For (**G**–**I**) statistics as in (**D**–**F**), * significant, 0.05 > *p* > 0.01; ** very significant, 0.01 ≥ *p* > 0.001; *** highly significant, *p* ≤ 0.001; n.s.—not significant.

The initial series of experiments was designed to replicate and extend earlier studies on the effects of CCCP on calcium activity in primary cultured hippocampal neurons [19]. The measurements in the current study were performed in the transfected neurons (PS1 and mPS1) and in adjacent non-transfected controls. Cultured neurons express spontaneous calcium transients (in most cases related to a network burst) in a manner that is similar in the transfected and control neurons (Figure 1), indicating that transfection by itself does not affect synaptic connectivity among neurons. The application of CCCP had a biphasic action, initially causing a rise in the frequency of the bursts, lasting about ten minutes, followed by a depression of activity and a concomitant increase in ambient cytosolic [Ca^2+^]_c_. In this respect, there was a striking difference between mPS1 and PS1 or non-transfected neurons (Supplementary Materials Videos S1 and S2) in that the former group raised [Ca^2+^]_c_ faster than the other two groups or non-transfected cells in the same dish (Figure 1A). The initial effect of CCCP involved an increase in the frequencies of the calcium transients in the control and PS1 but not in mPS1 (Figure 1B,C), followed by a decrease in this property in all groups. The amplitudes (Figure 1D,E) of the bursts increased in the control and mPS1 but not in PS1 for both transfected and non-transfected cells.

**Figure 2:**
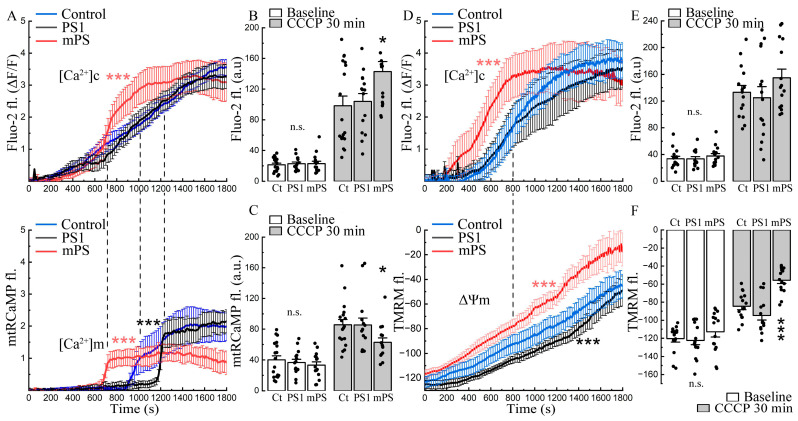
Effects of CCCP on cytosolic and mitochondrial calcium and mitochondrial potential in cells harboring mutant presenilin. (**A**) Cytosolic calcium (Fluo-2) rises faster in mPS1 cells (top panel), while mitochondrial calcium (mtRCaMP) rises sharply as cytosolic calcium increases (bottom panel). Pre-incubation with CCCP (10 μM) for 10 min, examples of recordings before treatment and with the initial stage of incubation are shown in Figure 1A–C. Control (Ct, *n* = 18 cells), PS1 (*n* = 15 cells), mPS1 (*n* = 15 cells), three cell cultures, DIV 10-14. Statistical comparisons from 600 to 1200 s on the graph (20–30 min of incubation), ANOVA, Fisher post hoc tests. (**B**,**C**) Averaged fluorescence values without normalization in control medium before incubation (baseline) and after 30 min incubation with CCCP for cytosolic and mitochondrial calcium, statistics as in A, ANOVA, Fisher post-hoc; F ([Ca^2+^]_c_/[Ca^2+^]_c_ + CCCP) = 0.11/3.94, F ([Ca^2+^]_m_/[Ca^2+^]_m_ + CCCP) = 0.52/2.97). (**D**) Averaged rise of cytosolic calcium (top panel, Fluo-2) and mitochondrial membrane potential (bottom panel, TMRM) with CCCP. Mitochondrial potential dissipation occurs more rapidly in mPS1 cells. Pre-incubation with CCCP for 10 min, statistical comparison of entire curves, *n* = 13 cells for each group, three cell cultures, DIV 10-14, ANOVA, Fisher post hoc tests. (**E**,**F**) Averaged fluorescence values without normalization in control medium before incubation and after 30 min incubation with CCCP for cytosolic calcium, F ([Ca^2+^]_c_/[Ca^2+^]_c_ + CCCP) = 0.46/1.33 and mitochondrial potential, F (Ψm/Ψm + CCCP) = 1.06/23.04, n = 15 cells for each group, three cell cultures, DIV 10-14, ANOVA, Fisher post hoc test, * significant, 0.05 > *p* > 0.01; *** highly significant, *p* ≤ 0.001; n.s.—not significant.

The time course of the effect of CCCP on the three groups of transfected cells is interesting indeed. While cytosolic calcium grows gradually after exposure to CCCP in the control cells and in the PS1 transfected ones, the mPS1 cells lead the rise (Figure 2A, top panel). Transfection delays the rise of mitochondrial calcium ([Ca^2+^]_m_, mtRCaMP) in the PS1 cells but speeds up the rise in the mPS1-transfected cells (Figure 2A, bottom panel). The mitochondrial calcium rises in mPS1 cells, associated with an early rise in cytosolic calcium that does not reach the levels of the control and PS1 cells, while the baseline calcium levels were similar (Figure 2C, white vs gray bars).

The change in mitochondrial calcium concentration is known to be linked to a change in mitochondrial membrane potential in that the calcium load depolarizes the mitochondrion and may impair its function [24]. In a series of experiments, we compared variations in mitochondrial voltage following exposure to CCCP, using TMRM as a sensor. While, in all cell groups, TMRM fluorescence indicated a gradual depolarization of the mitochondria from CCCP, the effect was significantly larger in the mPS1 group, and these cells were more depolarized than the other two groups, indicating a loss of function of the affected mitochondria (Figure 2D–F, white vs. gray bars).

**Figure 3:**
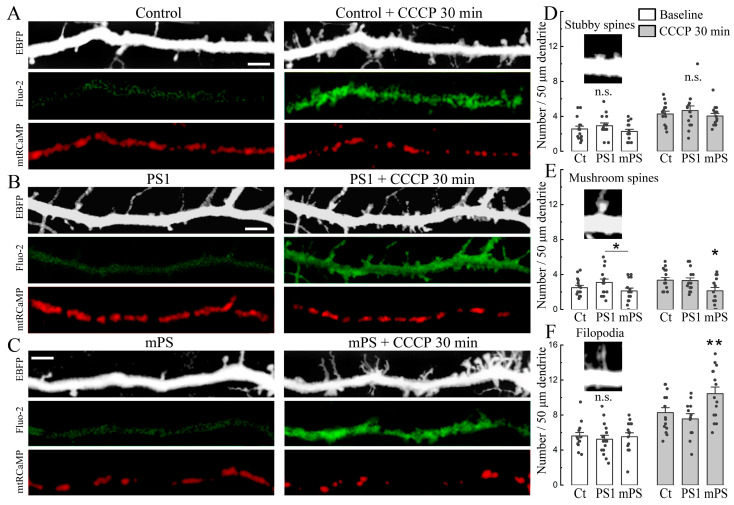
CCCP induces formation of filopodia during the rise of cytosolic calcium. (**A**–**C**) An example of dendritic segments of control (Ct), PS1, and mPS1 neurons before and after 30 min of exposure to 10 μM CCCP. Morphological marker (EBFP, monochrome), cytosolic calcium (Fluo-2, green), and mitochondrial calcium (mtRCaMP, red). Scale 5 μm. Note the large number of new filopodia after exposure to CCCP. Mitochondrial calcium for all cells is concentrated in a smaller area after exposure to CCCP. (**D**) Averaged number of individual stubby spines was compared in control conditions (baseline, F = 1.21) and after 30 min CCCP incubation (F = 0.65) per 50 μm dendrite length. (**E**) Averaged number of individual mushroom spines was compared in control (F = 2.18) and after 30 m CCCP incubation (F = 3.96) per 50 μm dendrite length. (**F**) Averaged number of individual filopodia was compared in control (F = 0.22) and after 30 m CCCP incubation (F = 5.3) per 50 μm dendrite length. The largest amount of new filopodia was observed in the group of mPS1. For (**D**,**F**) the equivalent number of dendritic segments was used for comparing cells, and the comparison was in the same dendrites, but there was no direct comparison among individual processes since they could change their morphology during exposure to the drug, *n* = 15 cells for each group, four cell cultures, DIV 10-14. ANOVA, Fisher post hoc test, * significant, 0.05 > *p* > 0.01; ** very significant, 0.01 ≥ *p* > 0.001; n.s.—not significant.

Prolonged exposure to CCCP produced a persistent rise in cytosolic as well as mitochondrial calcium. A morphological consequence of this rise is the formation of nascent filopodia and dendritic spines [19]. We compared the baseline as well as CCCP-induced spine formation in the control and PS1/mPS1 transfected neurons. In the analysis of these changes, we classified the new protrusions into stubby mushroom spines and filopodia (Figure 3). There was little or no difference among the groups in the baseline conditions (Figure 3D–F). The most striking effect of the drug was on the density of dendritic filopodia, and the largest change of the three groups was in the mPS1 cells (Figure 3C,F). This is consistent with the larger effects on [Ca^2+^]_c_ and [Ca^2+^]_m_ in the mPS1 cells, indicating that these cells have a limited effect on intracellular calcium levels compared to the control and PS1 transfected neurons.

**Figure 4:**
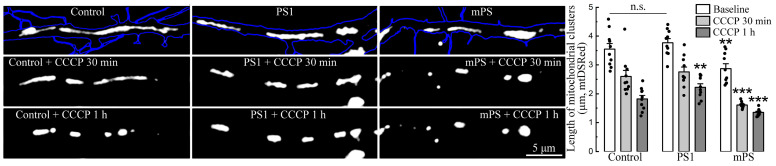
Mitochondrial morphology following CCCP. The length of mitochondria/mitochondrial clusters (monochrome, mtDSRed) in dendrites (blue, EBFP) was measured in control, PS1, and mPS1 cells in normal medium (baseline, F = 7.7), after 30 min (F = 16.09), and after 1 h of incubation with CCCP (F = 18.25). Mitochondria from mPS1 cells showed reduced length and became rounded after 30 min of incubation. Equivalent numbers of mitochondria in 1st and 2nd order dendrites were comparable, *n* = 10 cells for each group, three cell cultures DIV 10-14, ANOVA, Fisher post hoc tests, ** very significant, 0.01 ≥ *p* > 0.001; *** highly significant, *p* ≤ 0.001; n.s.—not significant.

Furthermore, mitochondrial morphology also differed among the groups. Initially, under baseline conditions, mitochondrial clusters of mPS1 cells already had a reduced length (2.87 ± 0.12 μm) compared to PS1 and the control (3.72 ± 0.14 and 3.51 ± 0.16 μm, respectively). Following exposure to CCCP, mitochondria of all groups were subjected to fission and rounding, but the greatest effect was seen after 30 min of treatment in mPS1 cells (Figure 4, examples and light gray bars), while after 1 h, the mitochondria of all groups were highly rounded.

We then examined mitochondrial motility in the dendrites of transfected neurons using morphological mitochondrial marker, mtDSRed. Before the CCCP treatment, mitochondrial motility did not differ significantly among the three groups in the anterograde (from the soma to the distal dendrites) and retrograde (towards the soma) directions. For control/PS1/mPS mitochondria, the speed of movement in the anterograde direction was 7.45 ± 0.86/6.66 ± 1.0/5.04 ± 0.88 SEM μm/min, in the retrograde direction 8.41 ± 0.83/5.78 ± 0.56/6.37 ± 0.56 SEM μm/min, respectively (Kruskal–Wallis ANOVA, Dunn’s post hoc tests, no significant differences for both directions). The percentage of the mobile mitochondrial pool in the dendrites of control conditions was 42 ± 3/41 ± 5/44 ± 4% for the control/PS1/mPS1, respectively, and also did not differ significantly among the three groups (Kruskal–Wallis ANOVA, Dunn’s post hoc tests). However, in a 1 h incubation with 10 μM CCCP, we observed an almost complete impairment of mitochondrial motility in the dendrites in both directions: 95 ± 1/95 ± 2/96 ± 2% for the control, PS1, and mPS1 mitochondria, respectively. There were no significant differences between the groups (Kruskal–Wallis ANOVA, Dunn’s post hoc tests, *n* = 10 cells for each group, the same as for Figure 4).

**Figure 5:**
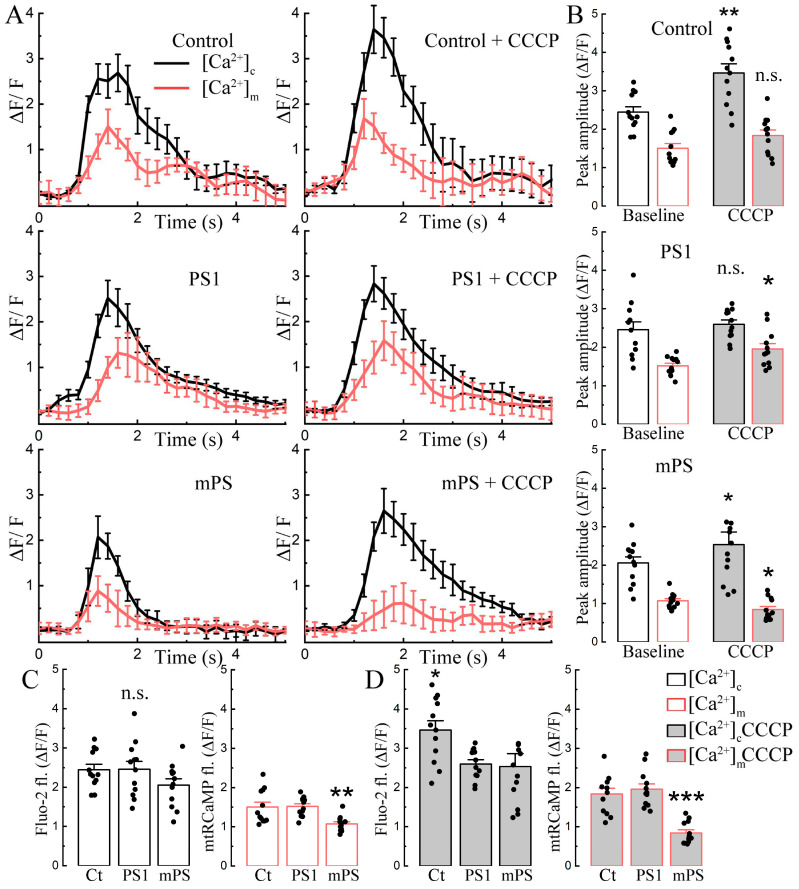
CCCP changes calcium activity differently in control and in mutant neurons. (**A**) Averaged calcium bursts (black trace, cytosolic calcium, [Ca^2+^]_c_) and associated mitochondrial calcium responses (red trace, [Ca^2+^]_m_) in control medium and during first 10 min of incubation with CCCP in control, PS1, and mPS1 neurons. Averaging was performed relative to the peak of the event in cytosolic calcium. (**B**) Averaged peak amplitude of cytosolic calcium burst (black) and associated mitochondrial calcium (red) in the control medium, and first 10 min incubation with CCCP (10 μm); *n* = 12 cells for each group, four cell cultures DIV 10–14, an equivalent number of calcium events was assessed for each cell, paired *t*-tests, *t*_control_ ([Ca^2+^]_c_/[Ca^2+^]_m_) = 3.66/1.8; *t*_PS1_ ([Ca^2+^]_c_/[Ca^2+^]_m_) = 0.11/2.22; *t*_mPS1_ ([Ca^2+^]_c/_[Ca^2+^]_m_) = 2.55/2.37. (**C**,**D**) Comparison of averaged peak amplitudes of cytosolic calcium events and associated mitochondrial calcium responses between groups before and after incubation with CCCP, statistics as in (**B**) ANOVA Fisher post hoc tests; F ([Ca^2+^]_c_/[Ca^2+^]_c_ + CCCP) = 1.86/4.6), F ([Ca^2+^]_m_/[Ca^2+^]_m_ + CCCP) = 8.26/24.6),* significant, 0.05 > *p* > 0.01; ** very significant, 0.01 ≥ *p* > 0.001; *** highly significant, *p* ≤ 0.001; n.s.—not significant.

In an attempt to explain the change in network activity produced by CCCP to other cellular properties, we compared cytosolic and mitochondrial calcium in the same neurons (Figure 5). The changes in the two calcium compartments were measured during network bursts. CCCP enhanced the cytosolic bursts of control cells, while the mitochondrial responses remained unchanged. No cytosolic differences were found in the treated PS1 group, while the mitochondrial response was slightly increased. Mutant cells in CCCP increased cytosolic bursts, similar to the control group, but significantly decreased mitochondrial transients (Figure 5A,B). Overall, [Ca^2+^]_c_ of mPS1 was dramatically reduced both before and after CCCP (Figure 5C,D).

The dynamics of the change in neuronal properties from CCCP was studied in the control and mPS1 groups. Based on results of the earlier experiments [12], we reasoned that we could focus on the differences between mPS1 and the controls in the current samples. Cells were patch-clamped in either voltage or current clamp modes. Their passive properties were first measured, followed by responses to a depolarizing ramp from -100 mV to +60 mV, and the change in mtRCaMP fluorescence was measured with a Prime Photometrcs (Oxfordshire, UK) camera at a high rate (20–100 frames/s). In additional experiments, miniature excitatory postsynaptic currents (mEPSCs) were measured in the presence of 0.5 μM tetrodotoxin (TTX) before and after exposure to CCCP. Cells were rejected if their passive properties deteriorated during the experiment.

**Figure 6:**
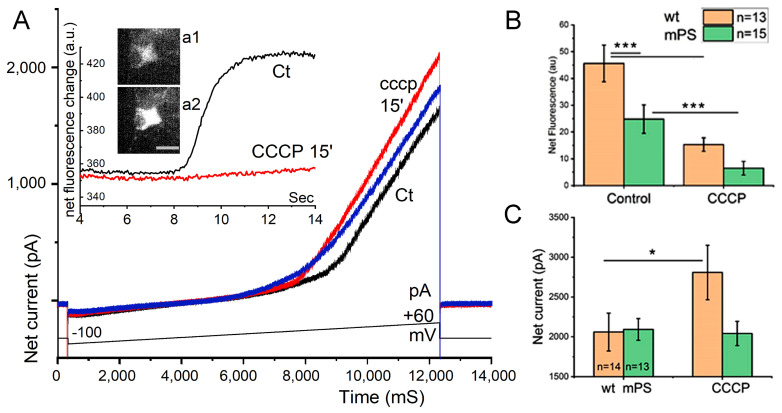
Patch-clamp recording from neurons transfected with GFP, mtRCaMP, and control or mPS1 plasmids. Recording was made in voltage clamp mode before and after application of CCCP. In the same neurons, somatic imaging at 488 (for morphology) and 550 (red, for mtRCaMP) was made (see Section 4). (**A**). Composite image of net fluorescence change seen in the soma of mPS1 transfected neuron (inserted images, before (a1) and at the end of the ramp (a2) associated with the current response to a depolarizing ramp between −100 and +60 mV (over 12.5 s, with resting membrane potential at appx. −60 mV). Insert: change in fluorescence level during the ramp showing that the control fluorescence change is much larger in the control than after CCCP. Sample illustration of the current responses to the voltage ramp are depicted in black (before), right after CCCP (red), and 15 min later (blue). Note that very low current passed through the membrane in the negative arm of the ramp (−100 to 0 mV), while the slope was much sharper in response to the depolarizing leg. Note also that the presence of CCCP caused a slight increase in the positive current response, and the effect faded in the 15 min test. The drug markedly suppressed the increased fluorescence seen in response to the ramp. Scale bar 10 μm. (**B**)**.** Net fluorescence changes in control and mPS1 neurons before and after exposure to CCCP. Note a significant difference between the two groups of neurons in both control condition and in presence of CCCP, *n* (Ct) = 13, *n* (mPS1) = 15 cells. (**C**). Net current change (between 0 and +60 mV) in control (Ct) and mPS1 cells before, left, and after exposure to CCCP. Note the lack of difference in net current change in control conditions and a large difference under CCCP, indicating that there is no difference between the two groups in baseline current change, while there is a significant difference in fluorescence change between the groups in baseline response significant differences between groups: *n* (Ct) = 14, *n* (mPS1) = 13 cells, four cell cultures DIV 10-14, *t*-tests, * significant, 0.05 > *p* > 0.01; *** highly significant, *p* ≤ 0.001. Scales (in (**C**)) 10 pA, 20 ms.

A depolarizing ramp in voltage-clamped neurons produced a fast (within 8–12 s) increase in mtRCaMP fluorescence that decayed over the following 5–10 s (Figure 6). This was associated with a large increase in outward current (Figure 6B,C), starting at or close to 0 mV potential. No conspicuous inward current was detected when the membrane potential was at rest or below the ramp. In addition, there was no persistent inward current when the cells were recorded in a bath containing TTX, while mPS1-transfected neurons produced a significantly smaller rise in mitochondrial fluorescence compared to control neurons (Figure 6). This contrasts with a similar increase in membrane currents in the two groups from the depolarizing ramp. This indicates that the smaller increase in mtRCaMP is not caused by a lower membrane current produced in the transfected neurons.

Upon exposure to CCCP, both the control and mPS1 groups significantly reduced their responses to the depolarizing ramp, with mPS1 neurons responding even less under the drug condition. This dramatically illustrates the effect of CCCP on mitochondrial function, as indicated above.

**Figure 7:**
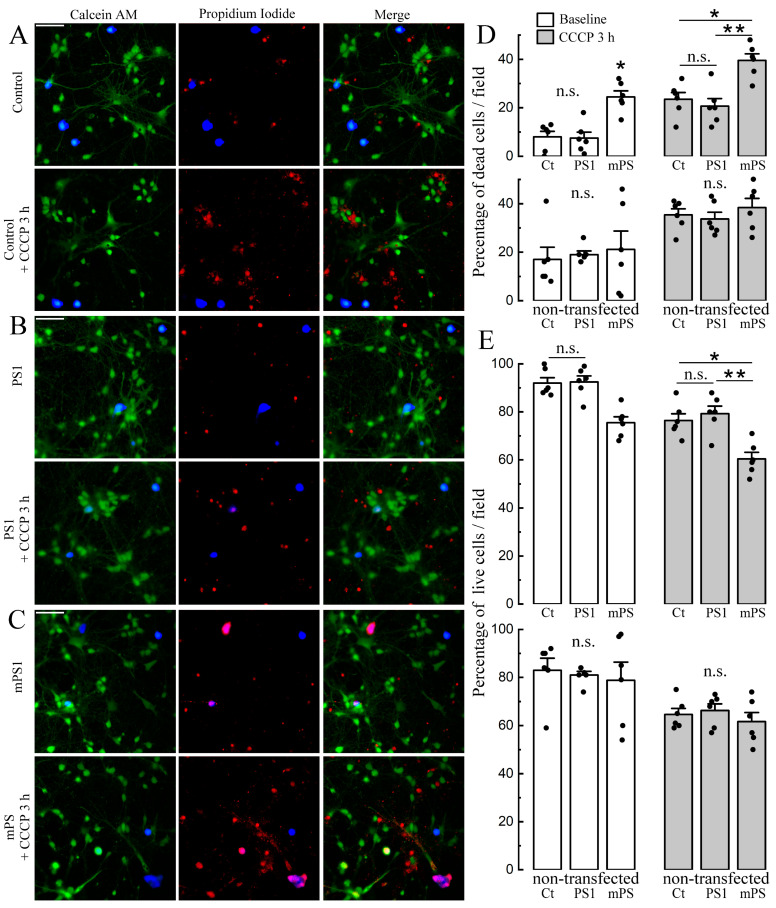
Comparison of the survival of control, PS1, and mPS1 cells before and after 3 h of incubation with CCCP using calcein and propidium iodide as apoptotic indicators. (**A**) An example of control transfected (blue, EBFP) cells on the background of non-transfected cells in normal imaging medium (top panels) and after 3 h of incubation with CCCP (10 μM, bottom panels) loaded with calcein AM (2 μM, green) and propidium iodide (2.5 μM, red). Scale bar 50 μm. (**B**) Same as for (**A**), for transfected PS1 and non-transfected cells. (**C**) Same as for (**A**), for transfected mPS1 and non-transfected cells. (**D**) Averaged percent of dead transfected (top) and non-transfected neurons (bottom) per field before (baseline), H (transfected) = 10.79, H (non-transfected) = 1.18 and after 3 h of incubation with CCCP (10 μM), H (transfected) = 10.6, H (non-transfected) = 1.14; white vs. gray bars. E. Averaged percent of live transfected (top) and non-transfected neurons (bottom) per field before (baseline), H (transfected) = 10.8, H (non-transfected) = 1.18 and after 3 h of incubation with CCCP (10 μM), H (transfected) = 10.6, H (non-transfected) = 1.13; white vs gray bars. (**D**,**E**) Values are mean ± SEM, *n* = 6 cultures for each group, DIV 10–15; Kruskal–Wallis ANOVA, Dunn’s post hoc tests, * significant, 0.05 > *p* > 0.01; ** very significant, 0.01 ≥ *p* > 0.001; n.s.—not significant.

We also analyzed the survival rate of cells exposed to CCCP in the three groups. For this, we used the live/dead ratio [19,25] to estimate the number of cells exposed to CCCP for three hours in both the control and transfected neurons. The results indicate a significant reduction in the number of live cells in the mPS1 group (Figure 7E) compared to the other groups. This difference was highly significant (*p* < 0.001). There was no apparent difference between PS1 and control cells and between control and non-transfected cells (Figure 7).

**Figure 8:**
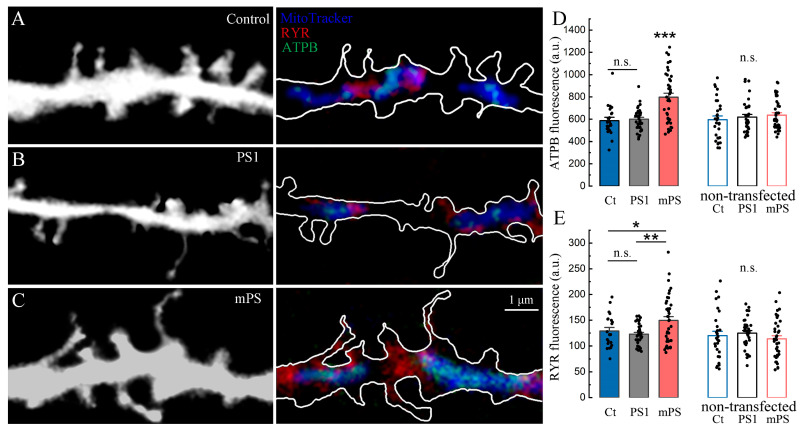
Colocalization of mitochondria, ATP synthase subunit beta (ATPB), and ryanodine receptors (RYR1), identified with immunocytochemistry. (**A**–**C**) Segments of dendrites of control, PS1-, and mPS1-transfected neurons, morphological marker EBFP (left panels—monochrome, on the right—white outline). MitoTracker (blue) was used to display mitochondrial morphology. The highest intensity of RYR1- (secondary antibodies anti-rabbit DyLight^®^ 650, red) and ATPB-related (Cy2 anti-mouse, green) fluorescence are colocalized with mitochondria in the dendrites of mPS1 neurons. Scale = 1 μm. (**D**) Averaging of ATPB fluorescence colocalized with mitochondria in dendrites in transfected neurons (F = 16.79) and in control adjacent non-transfected neurons (F = 0.61). (**E**) Averaging of RYR fluorescence colocalized with mitochondria in dendrites in transfected neurons (F = 5.57) and in control adjacent non-transfected neurons (F = 0.8). (**D**,**E**) Equivalent numbers of dendritic segments per cell were analyzed for each group, *n* (control/non-transfected) = 22/30 cells; *n* (PS1/non-transfected) = 35/35 cells; *n* (mPS1/non-transfected) = 40/40 cells; 10 DIV; ANOVA, Tukey’s post hoc tests, * significant, 0.05 > *p* > 0.01; ** very significant, 0.01 > *p* > 0.001; *** highly significant, *p* ≤ 0.001; n.s.—not significant.

We conducted an immunohistochemical study to detect the levels of beta-ATP synthase subunit (ATPB) in the control, PS1, and mutant presenilin groups. Initially, we verified the colocalization of ATPB with the mitochondrial tracker (Figure 8A–C). We found that the level of ATPB was significantly higher in the mitochondria of mutant cells (Figure 8D), and in addition, this increase was accompanied by the elevation of ryanodine receptor 1 (RYR1, ibid, E), typically localized near mitochondria.

Thus, we hypothesize that the vulnerability of mutant neurons, aggravated by treatment with CCCP (Figure 7), is based on disturbances in mitochondrial energetics initiated by the chronic dysregulation of calcium homeostasis due to presenilin dysfunction. We should emphasize that the dysregulation of ATPB and RYR levels associated with the mutated presenilin cells may be both the cause and the consequence of impaired calcium signaling.

## 3. Discussion

The present study compares the effect of CCCP, a selective mitochondrial uncoupler, on neuronal properties and the survival of mutant presenilin 1 (mPS1)-transfected neurons compared to PS1 and controls. As suggested in animal models and in humans, mPS1 plays a critical role in the formation of neural plaques associated with the emergence of the familial form of Alzheimer’s disease. While this is a common observation, there is still a paucity of information on the link between the formation of plaques, cell death, (a major hallmark of AD), and mPS1. It has been suggested that PS1 regulates [Ca^2+^]_c_ as well as the calcium concentration in endoplasmic reticulum and mitochondria (MT). There is an intuitive link between cell survival and [Ca^2+^]_c_; therefore, we now provide direct evidence for the effect of mPS1 on cell survival, at least within in vitro cultured hippocampal neurons.

Earlier studies by others [20,21] employed CCCP as a selective poison for MTs in relation to the development of AD. In one of them, memantine, a NMDA antagonist, was found to overcome the effect of CCCP on MTs [20], while in another study, CCCP affected mitophagy, a critical mechanism for restoring MT functions [21]. Interestingly, another recent study suggested [26] that CCCP can be used as a mitophagy promoter, which enhances cell survival. This could contradict our previous [19], current, and others’ [27,28,29] results. As reported in neurons, unlike the other cell types, mitochondrial depolarization caused by CCCP practically does not cause the recruitment of Parkin to the mitochondria [27] to trigger mitophagy. Another study reported that neuronal mitophagy is a much slower process, and when treated with CCCP, Parkin translocation between 0.5–6 h is rarely observed [30]. Moreover, the proportion of neurons in which Parkin translocation occurred did not exceed 30%, while treatment of HeLa cells with the same concentration of CCCP or SH-SY5Y cells with 20 μM CCCP can induce the association of Parkin with mitochondria in approximately 80% of cells for 1–2 h [30,31]. Obviously, some cell types are less dependent upon mitochondrial respiration. Neurons can rapidly initiate the process of apoptosis before Parkin translocation, especially in the setting of chronic mitochondrial dysfunction caused by mutant presenilin 1. An interaction between mPS1 and CCCP was not studied in any of the studies discussed above, including our earlier publication [19].

In an earlier study, we did explore the difference between PS1 and mPS1, and for this, we used paraquat [12] as a toxin. The current study replicated and extended these earlier results. We now added morphological changes in responses to CCCP, including enhanced filopodia formation in mutant cells (Figure 3) and a faster rounding of MTs (Figure 4). Moreover, in our experiments, along with the rounding and fragmentation of mitochondria against the background of the dissipation of their membrane potential, we observed an almost complete cessation of mitochondrial mobility in both the anterograde and retrograde directions in dendrites up to the end of the first hour of exposure to CCCP. Under such conditions, we cannot assert that the further translocation of mitochondria into the cell soma for degradation could have occurred [30], as this was not studied here, but we observed apoptotic signs in 20–40% of cells as early as three hours of incubation with the drug (Figure 7D). Thus, the fraction of motile mitochondria following CCCP did not exceed 4–5% in any direction. Nevertheless, it should be noted that different cell types and mitochondria may respond differently to CCCP, and the direction and magnitude of effect on mitochondrial voltage may depend on the age, cell type, timing, and concentration of the drug [32,33].

Several mechanisms regulate free intracellular calcium concentration, which is kept in the nM range but can rise to the high μM range. Two types of organelles, the endoplasmic reticulum (ER) and MT, are considered calcium stores that allow for an influx of calcium at higher concentrations on a fast time scale. The ER and MT are linked to allow a shift of excess Ca^2+^ from one compartment to the other. The regulation of calcium efflux into and out of mitochondria includes several molecular mechanisms that have been studied extensively in recent years, by us and others [34,35,36]. Among them is the Na/Ca exchanger that is controlled by NCLX to allow for an influx of calcium into the MT [37]. NCLX is accelerated by a blockade of PDE2. This sequence of events regulated the MT calcium concentration.

Recent hypotheses suggest that PS1 acts as a critical molecular link between the ER and postsynaptic mitochondrial clusters, regulating an efflux of calcium from local stores towards the narrow gap of MAM. One possibility is that PS1 functions as a leak channel from the ER. Alternatively, it modulates and enhances calcium leakage through ryanodine receptors [8,14]. Since the loading of the postsynaptic segment of the ER with calcium in each specific compartment depends on the activity of the entire synaptic machinery [34,38], it therefore can be assumed that PS1 is highly involved in the control of Ca^2+^ homeostasis inside the local mitochondria. In particular, in the present article, we demonstrate that mPS1 suppresses Ca^2+^ fluctuations in local postsynaptic mitochondria and reduces their potential under CCCP.

The role of mitochondrial calcium transients, related to the ongoing synaptic activity, is rather unclear. One may represent it as a messenger between the postsynaptic ER and the local mitochondrion, transferring signals through MAM in order to regulate ATP production, specially and temporarily associated with current energy requirements. If so, the reduced [Ca^2+^]_m_ responses in mPS1 may reflect the impaired ER–mitochondria communication (Figure 5). Furthermore, in accordance with the suggested hypothesis, the elevated levels of ATPB and RYR might reflect the attempt of the postsynaptic compartment to compensate for the lack of local MAM signaling. However, the raised cytotoxicity due to the overexpression of ATPB beta subunit is the other, negative side of such compensation.

In their turn, mitochondria are able to handle exceedingly high local calcium signals. We have recently suggested that synaptic mitochondria are capable of limiting the diffusion of Ca^2+^ inside the dendritic space to 2–5 μm. This mechanism may provide the specificity of the second messenger effects, which regulate the metabolic activity of MTs or even their compartments within a synaptic cluster [34].

We assume that the key role in the creation of “hot spots” on the surface of the dendrite, which serve as foci for the formation of actin protrusions and rapid growth of filopodia, is the store-operated calcium entry mechanism. In an earlier study, we suggested and recently demonstrated such a relationship [19,23]. This pathway is likely to trigger an early synapse formation during development. It may be no less important in the multiplication of existing contacts with the axon in synaptic plasticity [23]. An important question is related to what role mitochondria play in this and why filopodia particularly abundantly outgrow cells transfected with mPS1. We assume that this is due to the loss of control over the level of cytosolic Ca^2+^ under the influence of CCCP, which most quickly and most actively appears in the presence of the mutant (Figure 2 and Figure 3).

As highly dynamic structures, MTs move back and forth from the nucleus to remote dendrites and axons and are responsible for the supply of ATP to regions of high demand. The MTs may undergo a process of fusion, where two MTs fuse to form one large organelle. The opposite process is fission, where one large MT divides into two smaller structures [39]. This process allows for a better mobility of the MT through thin axons and dendrites. In the more extreme cases, MTs round up to form circular structures [40]. We have shown such changes following exposure to CCCP (Figure 4), and this is most likely associated with the prolonged rise in ambient calcium. As indicated above, there is a delicate balance between Na^+^ and Ca^2+^ exchange into and out of MTs. As it has been recently illustrated, a calcium load in mitochondria leads to an increase in Na^+^ concentration and, consequently, depolarization of the MTs and the loss of their function [41,42].

The main role of the MT is to provide ATP to cellular compartments in need. A primary consumer of ATP is the synapse [43]. While the link between the synapse and nearby MTs is not entirely clear, MTs are involved in the formation of synaptic connections. Surprisingly, an apparent reduction in MT functions, such as in the presence of CCCP, facilitates the formation of filopodia, the primordium dendritic spines. This is probably a consequence of a persistent rise in [Ca^2+^]_c_, as seen elsewhere [19]. Interestingly, mPS1, shown to cause a faster rise of [Ca^2+^]_c_ (Figure 1 and Figure 2), also causes a larger increase in filopodia density compared to the other cells (Figure 3). This increase in activity may underlie the earlier cell death seen after prolonged exposure to CCCP (Figure 7). This effect of mPS1, rather than the sheer formation of plaques seen later in life, is likely to underlie the progress of senility, the hallmark of AD.

The association of MT with AD has been the focus of attention in recent years. Obviously, mutated PS1 impair the function of MT and lead to the eventual formation of amyloid-*β* (A*β*) senile plaques. Consequently, a major effort has focused on the removal of the plaques, but this has not produced a breakthrough in the treatment of the disease. In addition, there were several illustrations of people with confirmed plaques but no cognitive deficits, and so, a direct link between plaques and AD is not yet so obvious. Another hallmark of AD has been the formation of tangles, formed by hyperphosphorylated tau proteins in neurons [44]. Since both processes develop in the brain over a long period of time, it is not clear if one leads to the other or vice versa. Suffice it to say that the removal of plaques by drug treatment does not cure the cognitive disease, but in both avenues of research, it is obvious that failures of MT calcium regulation are likely to trigger the formation of the disease. Moreover, the present results produce a direct link between MT, calcium regulation, and cell death. What is missing in this avenue of understanding AD is the ability to treat malfunctional MTs in order to overcome the lack of normal PS1.

Mitochondria are the main source of reactive oxygen species (ROS), which contribute to the pathogenesis of a number of neurodegenerative diseases, including AD [45]. ATP synthase activity in mitochondria can contribute to ROS production, and although not being a direct source of ROS, it can regulate energy metabolism and modulate pathways leading to ROS generation, cell death, and/or survival [46]. The amount of ROS production varies quite widely depending on the tissue type, and the specific endogenous mechanisms responsible for changes in neuronal mitochondrial ROS production are poorly studied compared to the other tissues [47]. Reportedly, the α subunit of ATP synthase was identified as being modified by carbonylation, nitration, etc., which was associated with a significant reduction in ATP synthase catalytic activity in the brains of AD patients [48]. Regarding the *β* subunit of ATP synthase, its upregulation in the brain of patients with AD and in triple transgenic AD mutant mice (3xTg-AD) has been shown [48,49,50]. The activation of the *β* subunit of ATP synthase may lead to the disruption of mitochondrial energy metabolism and the subsequent inhibition of ATP synthase activity [48]. Mitochondrial modulation can induce ROS production as a result of the dimerization of *β* subunits of F1Fo-ATP synthase, which probably contributes to the formation of the mitochondrial permeability transition pore, initiates permeabilization of the outer mitochondrial membrane and leads to the apoptotic death of neurons [50]. Thus, in our study, we confirmed that the level of ATPB was increased in the mitochondria of mutant cells (Figure 8), and in addition, this increase was combined with elevated levels of RYR near the mitochondria. These results confirm the link between MT, RYRs, calcium stores, and vulnerability to toxic insult, which will result in cognitive deterioration.

## 4. Materials and Methods

Cultures: Animal handling was performed in accordance with the guidelines published by the Institutional Animal Care and Use Committee of the Weizmann Institute and with the Israeli National guidelines on animal care. Cultures were prepared as detailed elsewhere [12]. Briefly, E17 rat embryos were removed from pregnant decapitated mother’s womb under sterile conditions, and their brains were removed; the hippocampi were dissected free and placed in a chilled (4 °C), oxygenated Leibovitz L15 medium (Thermo Fisher Scientific Inc., Waltham, MA, USA) enriched with 0.6% glucose and gentamicin (Sigma, St. Louis, MO, USA, 20 μg/mL). Tissue was mechanically dissociated with a fire-polished pipette and passed in the plating medium consisting of 5% heat inactivated horse serum (HS), 5% fetal calf serum (FCS), prepared in MEM-Earl salts (Biological Industries, Beit Haemek, Israel), enriched with 0.6% glucose, Gentamicin, and 2 mm glutamax. About 10^5^ cells in 1 mL medium were plated in each well of a 24-well plate onto polylysine-coated 13 mm circular glass coverslips. Cells were left to grow in the incubator at 37 °C, 5% CO_2_.

Plasmids: Neurons were transfected with PS1 or the M146V mutant presenilin 1 (mPS1), EGFP or EBFP (to image cell morphology), mtDSRed (to image mitochondrial morphology), and a mitochondrial calcium sensor (mtRCaMP) using lipofectamine 2000 (Thermo Fisher Scientific) at 1 μL per well with 50 μL per well Opti-MEM (Thermo Fisher Scientific) for 6–7 days in vitro (DIV) and were used for imaging at 10–14 DIV, depending on experiment. The transfection methodology was adopted from standard protocols [12]. It should be noted that, in all relevant experiments, parallel imaging was conducted with cells transfected with either PS1 or mPS1 and adjacent non-transfected controls. The basal fluorescence level was identical in the PS1 and mPS1 groups. In addition, transfection yield was similar in both groups (around 10–20% of the cells in a glass).

Live Cell Imaging: Cultures were incubated with Fluo-2 AM (2 μM, Thermo Fisher Scientific) for 1 h at room temperature to image variations in [Ca^2+^]_c_ resulting from network activity. Cultures were then placed in the 3 mL perfusion chamber on the stage of an upright Zeiss 880 confocal microscope using a 40× water immersion objective (1.0 NA). No photobleaching was detected under these conditions. Standard recording medium contained (in mM); NaCl 129, KCl 4, MgCl_2_ 1, CaCl_2_ 2, glucose 10, HEPES 10, pH was adjusted to 7.4 with NaOH and osmolality to 320 mOsm with sucrose. All measurements were conducted with identical laser parameters for all groups (e.g., intensity, optical section, duration of exposure, and spatial resolution) at room temperature.

Overall, each set of experiments was repeated on 3–6 different culture preparations, containing 24 separate cover-glasses, particularly for calcium/voltage mitochondrial measurements and spine morphology with 3–4 different preparations and for cell survival experiment—6 preparations.

Mitochondrial Membrane Potential: Cells were co-transfected with EBFP and PS1 or mPS1. Prior to the imaging, cells were incubated with tetramethylrhodamine methyl ester perchlorate (TMRM) (100 nM) for 20 min at room temperature. Mitochondrial potential was calculated using the formula developed by Koopman et al. [51]:

$$∆\Psi =-60\times \log\left(7.6\times \frac{F_{MT}}{F_{nuc}}\right)$$

Electrophysiology: Recording was made in standard conditions using patch pipettes containing (in mM) K-gluconate, 136; KCl, 10; NaCl, 5; HEPES, 10; ethylene glycol-bis (beta-amino ethyl ether) N,N,N′,N′-tetra-acetic acid (EGTA), 0.1; Na-GTP, 0.3; Mg-ATP, 1; and phosphocreatine, 5; pH 7.2 with an axis resistance in the range of 5–8 MΩ, as described before [12]. Signals were amplified using a MultiClamp 700B amplifier (Autom8, Berkeley, CA, USA) and accumulated and analyzed with pCLAMP 10 (Sunneyvale, CA, USA) software. Cells were recorded in either current or voltage clamp modes, and current responses to voltage ramp PS1 were recorded and analyzed for passive and active properties.

Assessing acute cell death, the dead/live assay: Cells were initially loaded in 3 ml standard recording medium with 2 μM calcein-AM in the presence of 2.5 μM propidium iodid (PI) at room temperature and imaged on a stage of a Zeiss LSM 880 confocal microscope using a 20× water immersion objective (1.0 NA). Two-channel images [Calcein AM/PI] were acquired, where green (488 nm) and red (545 nm) fluorescent cells represent live and dead cells, respectively. Percentage of live and dead cells was calculated as follows: live cells (cells stained green) or dead/dying cells (cells stained red) multiplied by 100% and divided by the total number of cells (green and red) in the field 593 × 593 micron at t = 0. Standard imaging software ImageJ (v.1.52 p, NIH, Bethesda, MD, USA) and Amira Software (Amira 3D 1 January 2023, Thermo Fisher Scientific) were used to count the number of live cells for each image.

Immunostaining: Cover glasses bearing transfected primary hippocampal cells 10 DIV were washed briefly with a standard extracellular solution and incubated with 200 nM MitoTracker^®^ Red CMXRos (Thermo Fisher Scientific) for 20 min. Then cultures were washed and fixed with 4% paraformaldehyde in 0.1 M phosphate-buffered saline (PBS, pH 7.4) for 20 min and washed thereafter with PBS thoroughly. Cultures were incubated for 1.5 h with 20% normal horse serum (NHS) in 0.2% Triton X-100 containing PBS and subsequently incubated overnight at 4 °C with the primary antibodies anti-ATPB mouse monoclonal (Abcam plc, Cambridge, UK, 1:500, gift from prof. Atan Gross, WIS, Rehovot, Israel) and rabbit anti-RYR1 antibody (Abcam, 1:100). Cultures were incubated for 1.5 h with Cy2 anti-mouse 1:250 and Donkey Anti-Rabbit IgG H&L 1:250 (DyLight^®^ 650), (both Abcam). Coverslips were rinsed, transferred onto glass slides, and mounted for visualization on a Zeiss (Oberkochen, Germany) upright LSM 880 (which allows simultaneous visualization of four fluorophores) with an anti-fading mounting medium using a Plan-Neofluar 40×/1.30 oil immersion objective. Image stacks were captured with identical laser parameters for all groups (e.g., intensity, optical section, and spatial resolution).

Fluorescence and statistical analysis: High-resolution fluorescent images were analyzed using ImageJ (v.1.52 p, NIH, Bethesda, MD, USA), Amira Software (Amira 3D 1 January 2023, Thermo Fisher Scientific), and MATLAB (R2010b, MathWorks, Inc., Natick, MA, USA)-based programs. Statistical comparisons were made with using MATLAB, KaleidaGraph (v. 4.5, Synergy, Inc., Reading, PA, USA), and Origin Pro 2021 (9.8.0.200, Electronic Arts, Inc., San Mateo, CA, USA) software.

The number of culture preparations was the statistical unit for survival/death experiment. In other cases, a particular property of a specific cell was the unit. Among parameters tested, there were number of spines/filopodia, calcium transients, and a set of parameters related to MT clusters. The single cell-related measurements were close to equal and were averaged with the mean value taken as *n*. This set of ns was tested for normality of distribution using Kolmogorov–Smirnov or Lilliefors test (for smaller samples). One-way ANOVA or Kruskal–Wallis one-way ANOVA and/or paired *t*-test were used depending on the particular task. Statistically significant differences were considered at *p* < 0.05; * significant, 0.05 > *p* > 0.01; ** very significant, 0.01 > *p* > 0.001; *** highly significant, *p* ≤ 0.001; n.s.—not significant.

## Data Availability

Data is contained within the article and Supplementary Materials.

## Supplementary Materials

The following supporting information can be downloaded at: https://www.mdpi.com/article/10.3390/ijms25010578/s1.
