# Supplementary material for: Cultured Rat Hippocampal Neurons Exposed to the Mitochondrial Uncoupler Carbonyl Cyanide Chlorophenylhydrazone Undergo a Rapid, Presenilin-Dependent Change in Neuronal Properties
Source: Int J Mol Sci. 2024 Jan 1;25(1):578. doi: 10.3390/ijms25010578 (PMC10779238; doi:10.3390/ijms25010578)
Supplement: Supplementary file 1 [file ijms-25-00578-s001.zip › Captions.pdf]

Supplementary video S1. Effect of CCCP on the network bursts of the mPS1 and control hippocampal neurons. Continuous time-lapse imaging of network burst (Fluo-2, green) in EBFP-transfected mPS1 neurons and control non-transfected neuron before and in the presence of CCCP (marked) was performed during 50 min at the video-rate of 50 frames/s. In the video file, 1 min = 1 s. Frame size is 420 x 420 μm. Note that cytosolic calcium in the top mutant neuron rises earlier than in control neurons. The beginning and end of the video contain the morphology (blue) of two mPS1 transfected neurons. By the end of the recording, the bottom mutant neuron with low calcium level shows damaged morphology, however, in terms of $\Delta F/F$ the bottom mutant neuron also raises cytosolic calcium.

Supplementary video S2. Effect of CCCP on network bursts of the PS1 and control hippocampal neurons. Continuous time lapse imaging of network burst (Fluo-2, green) in EBFP-transfected mPS1 neurons and control non-transfected neuron before and in the presence of CCCP (marked) was performed during 47 min at the video-rate of about 50 frames/1 s. In the video file, 1 min = 1 s. Frame size is 420 x 420 μm. Note that cytosolic calcium in PS1 neurons increases at the same rate or even with some delay compared to control neurons. The beginning and end of the video contain the morphology (blue) of two PS1 transfected neurons. By the end of the recording, both neurons show normal morphology.
